# Cost-Effective Technologies to Study the Arctic Ocean Environment †

**DOI:** 10.3390/s18072257

**Published:** 2018-07-13

**Authors:** Viviana Piermattei, Alice Madonia, Simone Bonamano, Riccardo Martellucci, Gabriele Bruzzone, Roberta Ferretti, Angelo Odetti, Maurizio Azzaro, Giuseppe Zappalà, Marco Marcelli

**Affiliations:** 1 Laboratory of Experimental Oceanology and Marine Ecology University of Tuscia, Molo Vespucci, Porto di Civitavecchia, 00053 Civitavecchia (RM), Italy; v.piermattei@unitus.it (V.P.); simo_bonamano@unitus.it (S.B.); riccardo.martellucci@unitus.it (R.M.); marcomarcell@unitus.it (M.M.); 2 Centro Euro-Mediterraneo sui Cambiamenti Climatici (CMCC), Via Augusto Imperatore, 16, 73100 Lecce, Italy; 3 CNR-ISSIA, National Research Council, Institute of Intelligent Automations Systems, Via de Marini, 6, 16149 Genova, Italy; gabriele.bruzzone@ge.issia.cnr.it (G.B.); roberta.ferretti@ge.issia.cnr.it (R.F.); angelo.odetti@ge.issia.cnr.it (A.O.); 4 CNR-IAMC, National Research Council, Institute for Coastal Marine Environment, Spianata S. Raineri, 86, 98122 Messina, Italy; maurizio.azzaro@iamc.cnr.it (M.A.); giuseppe.zappala@iamc.cnr.it (G.Z.)

**Keywords:** Arctic Ocean, low-cost technology, temperature, fluorescence of chlorophyll *a*

## Abstract

The Arctic region is known to be severely affected by climate change, with evident alterations in both physical and biological processes. Monitoring the Arctic Ocean ecosystem is key to understanding the impact of natural and human-induced change on the environment. Large data sets are required to monitor the Arctic marine ecosystem and validate high-resolution satellite observations (e.g., Sentinel), which are necessary to feed climatic and biogeochemical forecasting models. However, the Global Observing System needs to complete its geographic coverage, particularly for the harsh, extreme environment of the Arctic Region. In this scenario, autonomous systems are proving to be valuable tools for increasing the resolution of existing data. To this end, a low-cost, miniaturized and flexible probe, ArLoC (Arctic Low-Cost probe), was designed, built and installed on an innovative unmanned marine vehicle, the PROTEUS (Portable RObotic TEchnology for Unmanned Surveys), during a preliminary scientific campaign in the Svalbard Archipelago within the UVASS project. This study outlines the instrumentation used and its design features, its preliminary integration on PROTEUS and its test results.

## 1. Introduction

Increasing efforts to study and monitor the environment, especially in the Polar regions, call for data acquisition at higher temporal and spatial resolutions. This challenge can be met if new technologies designed for research in harsh environments are developed. This paper describes the new probe ArLoC (Arctic Low-Cost probe), specifically designed for this purpose, together with preliminary results obtained during a scientific campaign carried out in summer 2017 in the Svalbard Archipelago using the robotic system PROTEUS (Portable RObotic TEchnology for Unmanned Surveys).

Over the last few decades, polar oceans are the locations that have been affected most strikingly by global climate change, with temperatures and acidities changing at a rate that is twice the global average [1]. This has taken place in an already complex system of climatic phenomena that has led to a drastic decrease in Arctic Sea Ice (approximately a 40% reduction in the last decade), which is foreseen to result in its rapid disappearance [2]. The ongoing retreat of Arctic sea ice has been exposing increasingly large surface areas of the basin to sunlight, thus promoting increased growth of phytoplankton during the summer months [3] and causing phytoplankton communities and biomass changes [4,5]. In this respect, the Svalbard Islands represent a border area between the Atlantic and Arctic biogeographic zones, where the input of glacial freshwater and sediments create environmental gradients of suspended as well as dissolved matter and nutrient concentrations, inducing significant changes in community composition [6,7,8]. The Svalbard Islands can also serve as an optimal Arctic research environment to observe ecological changes thanks to the international polar research base at Ny-Ålesund [9]. Though the Arctic Ocean is considered to be a key indicator of climate change, it is poorly studied, and significant gaps still remain in the Global Ocean Observing System.

Technological advances in oceanographic measurement capabilities would be fundamental for observing and monitoring changes in marine ecosystems, particularly in remote regions. The implementation of remote oceanographic observatories in polar areas is a huge challenge: Device deployment is arduous; measurements near the ice fronts are hazardous [10]; shifting ice and icebergs make it difficult to deploy sea surface equipment over long periods and restrict access for research vessels [11]; marine conditions are often threatening and can cause damage or loss of equipment; surveys and probes are expensive, as is power supply required for autonomous applications and data communication [12,13].

In this light, the development of extended marine monitoring systems based on wireless and autonomous equipment will make it possible to collect data for calibration and validation of satellite images and mathematical models, thus helping us to describe and forecast ecological processes [14].

For all these reasons there is a definite need for accurate, continuous environmental monitoring based on cost-effective sensors for remote usage [15]. Low-cost, easily adaptable instruments would considerably reduce the cost of oceanographic surveys, as they improve data coverage to study the marine environment in greater detail [13,16,17] and increase repeatability. The use of these alternative technologies also enables researchers to provide sea-truth data for satellite observations, which are fundamental to study the marine environment at high spatial and temporal resolution but requires observational data for calibration and validation [18]. Continuous advances in sensor technology and materials make it possible to develop new probes with suitable characteristics for different oceanographic applications, providing new opportunities for global ocean observation and monitoring [19].

In this framework, our flexible, miniaturized ArLoC probe was designed for easy integration into various types of platforms, enabling continuous measurement of temperature, pressure, fluorescence of chlorophyll *a*, pitch, roll and acceleration. This study reports the preliminary results of its utilization within the UVASS (Unmanned Vehicles for Autonomous Sensing and Sampling) research project in the Svalbard Archipelago, and its integration onboard the PROTEUS vehicle. The goal of the UVASS project is to perform air, water and ice data sensing and sampling in dangerous areas close to marine glacier fronts using UMVs (Unmanned Marine Vehicles), i.e., ROVs (Remotely Operated Vehicles), AUVs (Autonomous Underwater Vehicles), USVs (Unmanned Surface Vehicles), USSVs (Unmanned Semi-Submersible Vehicles) and UAVs (Unmanned Aerial Vehicles) [20].

This paper is structured as follows: Section 2 reviews the state of the art of principal oceanographic instruments, and describes the technologies developed and used for this study as well as performed testing; Section 3 discusses the application of the technologies and data acquired during the survey. Finally, Section 4 discusses results and technology performance as well as future prospects.

## 2. Technology Description

### 2.1. State of the Art of Marine Probes and Sensors

Marine instruments for both physical and bio-optical measurement are extremely varied. Measurement ranges, resolution needs and technical features define and limit the operational capability of each sensor and probe. Extended marine monitoring would require a reduction in the cost of platforms and instruments, but this often prejudices measurement accuracy and consequently data quality. Thus, selecting an instrument depends on factors such as research and monitoring objectives, local characteristics and installation type [12].

Since marine instrumentation is usually very expensive, researchers are induced to develop systems based on open source and low-cost boards like, for instance, Arduino [13,21], in order to create extended networks made of multiple flexible nodes connected to each other to meet the various requirements of marine monitoring [22].

As the cost of commercial platforms may exceed $100,000 [23], considerable effort has been devoted to the development of low-cost buoys: The SEMAT network (Smart Environmental Monitoring and Analysis Technologies) promotes an inexpensive, flexible coastal system based on low-price technologies, totaling $2000 for a basic buoy without sensors or probes [16]. Numerous initiatives and projects have promoted a similar approach by developing heterogeneous remote and wireless networks for water quality monitoring [22,23,24].

The development of low-cost platforms has resulted in the design of a number of inexpensive though reliable sensors to be integrated into these observatory networks. Examples of commercial probes for oceanographic temperature measurements include: SBE 37 MicroCAT, an expensive probe whose cost is justified by very high-resolution sensors; YSI 600OMS, a moderately expensive multiparametric probe equipped with a lower quality temperature sensor; Idronaut Ocean Seven CTD, a high sensitivity temperature sensor with a cost comparable to the previous one. Regarding chla fluorescence sensors, it is very difficult to find a relatively low-cost sensor (below 1000 €); commercial sensors generally range between 1000 € and 5000 € (Cyclops-7F Turner Design, ECO FL Wetlabs, SeaPoint, UniLux Chelsea, MicroFlu TriOS); some more expensive instruments range between 5000 € and 10,000 € (EXO1 YSI); some may reach 10,000 € and over [25]. On the other hand, extremely low-cost commercial sensors can also be found, but these are characterized by very low resolution, which is inadequate in most marine applications. 

Table 1 summarizes the main specifications of the above probes and sensors.

### 2.2. The ArLoC Probe

**Figure 1:**
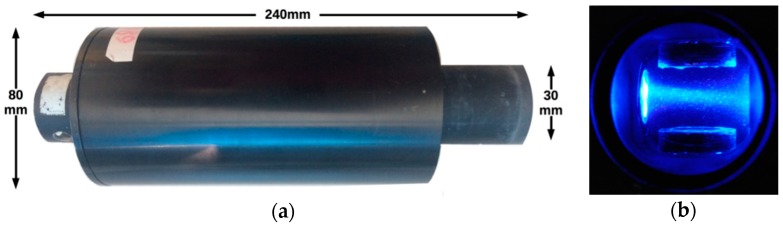
(**a**) ArLoC probe dimensions; (**b**) internal view of the measuring chamber with the excitation light source of chla fluorescence sensor.

The ArLoC (Arctic Low-Cost probe) is a miniaturized, low-cost, flexible probe (Figure 1) which was designed and constructed for easy integration into various kinds of oceanographic platforms. The probe derives from a modular, low-cost technology developed by Marcelli [26] but it was greatly improved in terms of electronic interface and supply. It is equipped with depth, temperature and fluorescence of chlorophyll *a* (chla) sensors, and with pitch, roll and acceleration sensors to control the behavior of the instrument. This probe is based on an in-depth study of inexpensive, high sensitivity components, seeking to obtain a product which could be at the same time sustainable and comparable with commercial probes. As a matter of fact, the cost of the ArLoC, equipped with all its sensors, is around 1800€, thus much lower than other commercial devices: a standard marine fluorometer usually ranges between 2000€ and 9000€ depending on accuracy, while a multiparametric probe can easily reach 20,000€.

**Figure 2:**
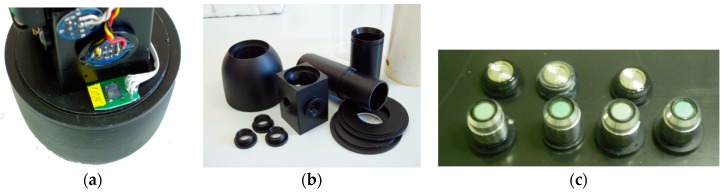
(**a**) The triaxial accelerometer installed on the bottom of the electronics; (**b**) ArLoC modular mechanical components; (**c**) sensors bushings, which can contain various kinds of sensor components.

The ArLoC is composed of a 240 mm long flow-through system. Its sensors are protected inside the measuring chamber, which has an internal diameter of 30 mm and is made of aluminum and carbon fibers to combine lightweight (<1 Kg) and resistance. The chamber hosts the main sensors: the temperature sensor is a spherical glass bulb thermistor with a resolution of 0.01 °C and 0.05 ms response time; the depth sensor is a Keller pressure transducer in the range 0–500 dbar; it is constituted by a wide, sensitive stainless steel membrane and is positioned inside the measurement cell, parallel to the water flow; it is powered at constant voltage and measured by a differential amplifier followed by a low-pass filter for noise reduction; the fluorescence of chla has a minimum detection limit of 0.01 µg/L and is composed of two modules: One module includes a LEDs light sources, pulsed at 512 Hz, which ranges between 430 and 470 nm, the other module is composed of a high accuracy photodiode covered by a red band-pass filter catching all the light between 660 and 720 nm, characteristic of chla emission. Moreover, a SCA3000 Series 3-axis accelerometer was fixed to the bottom of the ArLoC interior, in order to study the dynamic behavior of the probe (Figure 2a).

A key feature of the chla fluorescence sensor, in addition to low-cost and high sensitivity, is that it operates in a dark chamber and not externally like commercial probes. This prevents the ArLoC from solar radiation exposure, in particular from red light; many commercial sensors are affected by the negative influence of solar radiation reflecting from the marine surface.

The system’s modularity allows sensors and components to be changed inside the measurement chamber, enabling probe customization to fit specific needs (Figure 2b,c).

**Figure 3:**
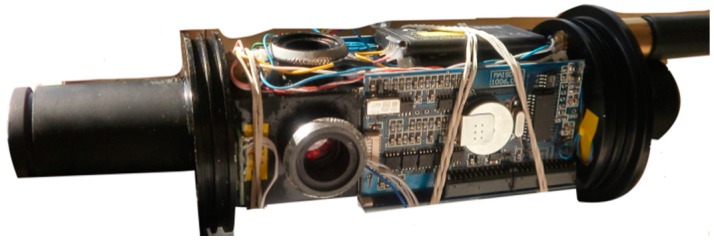
Electronic boards and components assembled around the measuring cell.

A Microchip PIC16F876-20 microcontroller was used. It is characterized by a high-performance RISC architecture, low power consumption, 8K flash program memory (14 bit words), 368 bytes of data memory, 256 bytes of EEPROM data memory, 13 interrupts, 3 I/O ports, 3 timers, serial communication interface, 5 × 10-bit A/D input channels. Data transmission is performed by an RS-485 serial interface with a baud rate of 19,200 bps, and a data frequency of about 5.6 Hz. Data can be acquired on a pc through a serial RS485/232 converter or stored in an internal memory and can be easily programmed through the RS485 serial port by an external device such as PROTEUS. Power supply, between 11 and 14 VDC > 100 mA (depending on the LEDs intensity), is given by the external platform; the ArLoC can also be equipped with two rechargeable lithium batteries for autonomous acquisitions. All the electronics boards were designed and built to be miniaturized as much as possible and were positioned all around the measuring chamber (Figure 3).

**Figure 4:**
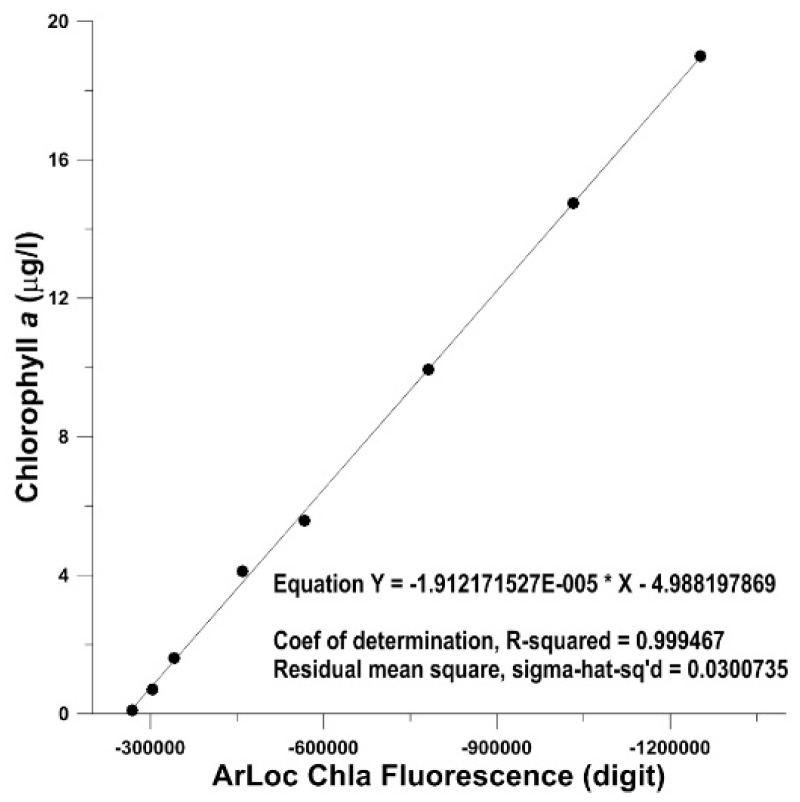
Linear regression between laboratory spectrophotometric chlorophyll *a* concentration and ArLoC chla fluorescence.

All the ArLoC sensors were calibrated in the laboratory with standard instruments. In particular a controlled flowing calibration chamber equipped with a Micro Thermosalinograph SBE 45 (Sea-Bird Electronics, Bellevue, WA, USA, temperature resolution: 0.0001 °C) and a FIAlab fluorometer (FIAlab Instruments Inc., Seattle, WA, USA, spectral range 310–750 nm) was specifically assembled. A 6-points temperature calibration curve was performed by varying the temperature of the bath in the range 0–30 °C with a thermocryostate Haake Thermo Scientific DC10/K10. The calibration of the ArLoC chla fluorometer was obtained using 8 phytoplankton solutions at increasing chla concentrations (range 0–20 µg/L); it was also analyzed with standard spectrophotometric methods [27,28] (Figure 4). Thanks to this procedure, it was possible to obtain chla concentration from ArLoC measurements, and in doing so avoiding using the samples collected using the automatic water sampler installed on PROTEUS that were needed for microbiological analyses.

### 2.3. PROTEUS Platform

**Figure 5:**
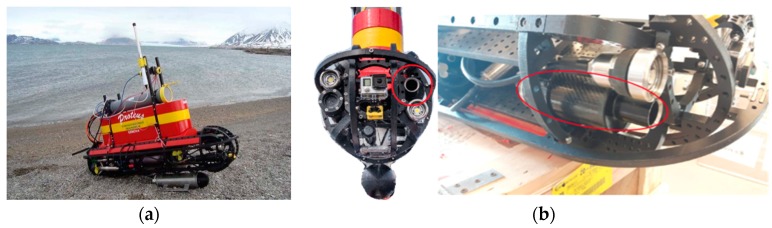
(**a**) PROTEUS vehicle; (**b**) front and side view of PROTEUS (circle shows ArLoC installed on board).

The ArLoC probe was integrated into an autonomous platform, PROTEUS (Portable RObotic TEchnology for Unmanned Surveys) (Figure 5a,b), which is an innovative unmanned marine vehicle designed, built and developed by the Field and Interaction Robotics research group at the ISSIA Institute, National Research Council, Italy. 

It is a portable (45–60 kg weight, 1.5 m × 0.35–0.5 m × 0.35–1.5 m LWH), highly modular and reconfigurable vehicle which is able to operate as an ROV, an AUV or a USSV. It was expressly designed for polar operations as it is based on the P2-ROV [29]. One of its main features is its open-frame chassis which is especially designed for quick installation and shifting of tools, equipment and sensors. Its thrusters are also interchangeable, displaceable modules. In this way the vehicle can be reconfigured upon each expedition according to specific needs. Moreover, thanks to its open hardware and software architectures, PROTEUS can easily be fitted on board with a number of different sensors and actuators.

During the scientific campaign performed in summer 2017, it was equipped with manifold sensors and samplers, i.e., the ArLoC and the Idronaut 305 Plus CTD probes, a Micron echo-sounder, a Cyclops-7 Turner Design turbidimeter, an automatic water sampler developed by CNR-IAMC [30] and surface and underwater video cameras, which were used to perform water data collection in the stretch of sea facing the Kronebreen and Blomstrandbreen glaciers. The Idronaut 305 plus was used in order to collect data and compare low-cost technology performance with ‘state of the art’ sensors. A data-logger allowed the reconstruction of PROTEUS’s path and behavior by acquiring GPS position and data from navigation sensors. In this way all the data collected were georeferenced and synchronized in time.

## 3. Data Acquisition and Analysis

### 3.1. Campaign Description

As mentioned above, an experimental survey was carried out during summer 2017 in the Kongsfjorden area in the Svalbard Archipelago in order to acquire data and water samples and to test and assess the capacity of new low-cost, portable technologies to acquire reliable data in an extreme environment. During the campaign PROTEUS was mainly used as an USSV and remotely controlled by means of a Wi-Fi radio link, a control station (command console) and a guidance camera mounted on top of the vehicle. During acquisitions, the vehicle was deployed and operated by personnel on board a small (31 ft) work boat (MS Teisten), which was kept at a safe distance from the fronts of the tidal glaciers located in the Kongsfjorden.

**Figure 6:**
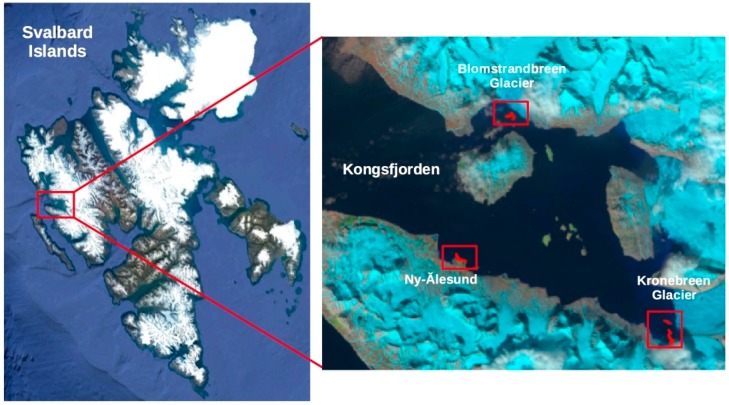
Study area and transects trajectories performed by PROTEUS vehicle.

In order to assess the achievement of design requirements and the performance of the instruments a series of transects were performed in three different areas between 18th and 23rd June 2017: Ny-Ålesund, Kronebreen glacier and Blomstrandbreen glacier, as shown in Figure 6. In this study the data acquired in front of Blomstrandbreen Glacier were analyzed in order to evaluate the effects of glacier melting on phytoplankton biomass and its distribution in the upper layers during the spring bloom period.

### 3.2. ArLoc In Situ Testing

**Figure 7:**
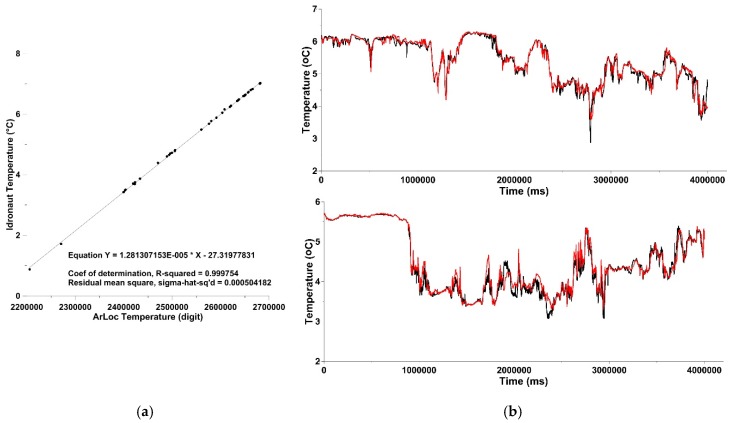
(**a**) Comparison between Idronaut 305 plus and ArLoC temperature sensors; (**b**) temperature trend acquired during two representative transects along Blomstrandbreen glacier, red for ArLoC data and black for the Idronaut 305 Plus data.

Data acquired by the instruments were processed in order to compare the newly developed technology with commercial sensors chosen as a benchmark. The comparison between temperature data measured by ArLoc and Idronaut 305 plus shows a very satisfactory correlation between the new technology and the standard probe, with a coefficient r^2^ = 0.999, proving the capacity of ArLoc to detect the temperature variations with high accuracy (Figure 7a).

Figure 7b shows the temperature trend recorded in two illustrative transects along the Blomstrandbreen glacier. ArLoC profiles consistently overlap with the Idronaut 305 plus ones, demonstrating accurate dectection of colder water masses due to the ice melting process across the area. Along the transects the values ranged between 2.87 and 6.29 °C with the minimum peaks detected in the proximity of the glacier.

**Figure 8:**
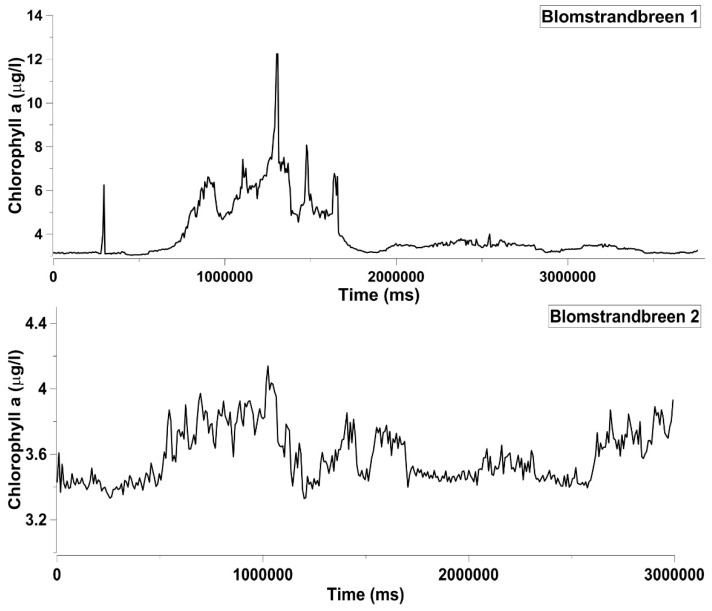
Chlorophyll *a* trend acquired by ArLoC along Blomstrandbreen 1 and Blomstrandbreen 2 transects.

Chlorophyll *a* data recorded in two transects along Blomstrandbreen Glacier exhibit median values of 8 µg/L. Chlorophyll *a* trend in the Blomstranbreen 1 transect shows an increase in concentrations moving closer to the ice, where the values reached a peak of 12 µg/L; as the distance from the glacier front increased, lower and more homogeneously distributed chla values were observed, as shown in the Blomstranbreen 2 transect (Figure 8). More generally, all transects exhibit high levels of phytoplankton biomass, in agreement with results obtained in previous investigations conducted in Kongsfjorden, which confirmed the occurrence of the spring bloom in the same period [7,31,32,33].

ArLoC data were also processed in order to obtain a representation of isosurfaces through the ODV (Ocean Data View) open source software package, which displays oceanographic, geo-referenced and trajectory data [34].

**Figure 9:**
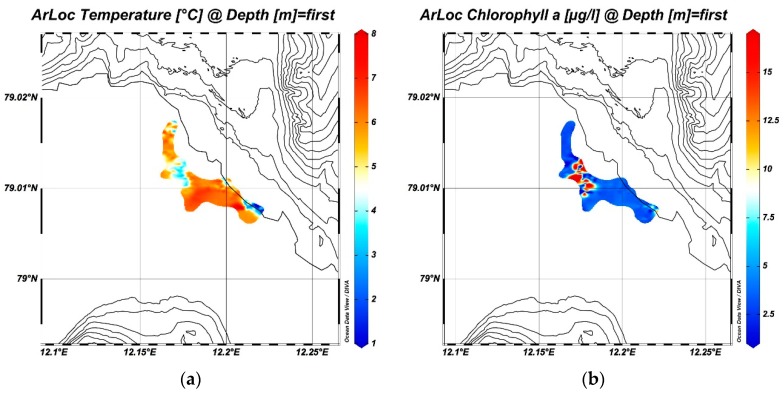
(**a**) Isosurface of temperature acquired by ArLoC along the Blomstrandbreen glacier; (**b**) chlorophyll *a* isosurface along the same transects.

Figure 9a,b show surface temperature distribution and chla obtained by integrating all the transects acquired by ArLoC along the Blomstrandbreen glacier. The temperature distribution maps highlight the influence of ice melting on coastal waters, proved by the presence of colder water masses (1–2 °C) in the areas bordering the glacier. Moreover, the maps show the effect of the water temperature on phytoplankton biomass, which reaches the highest concentrations (>10 µg/L) at the lowest temperatures: melting summer ice is a source of nutrient enrichment for coastal waters and stimulates the growth of marine phytoplankton, which reaches the high concentrations typical of a blooming event.

### 3.3. Application of ArLoC as Sea-Truth of Remote Sensing

The ArLoC probe’s realiability as sea-truth for satellite observations was also tested. Data were processed to compare ArLoC chlorohyll *a* acquisitions with a Level-1C Sentinel-2 product. Sentinel-2 images have a resolution of about 30 m and are acquired, processed and generated by the European Space Agency (ESA) and repackaged by USGS (U.S. Geological Survey’s) [35]. Sentinel-2 houses a Multispectral Instrument (MSI) which measures the reflected radiance in 13 different spectral bands. These data were acquired on the same day the survey was held and were processed by Acolite, the processing software developed at RBINS [36] both for Landsat and Sentinel data. This software executes the atmospheric correction and produces remote sensing reflectance (Rrs) for the available wavelengths. In this study, it was used to output the 560 and 664 band Rrs which is needed for applying the algorithm by Tzortziou et al. [37], based on the blue-green spectral band ratios and parametrized with the data collected in various coastal areas [36].

In order to test ArLoC’s ability to validate high resolution satellite observations, the normalized chla concentration from both probe and Sentinel-2 were compared.

**Figure 10:**
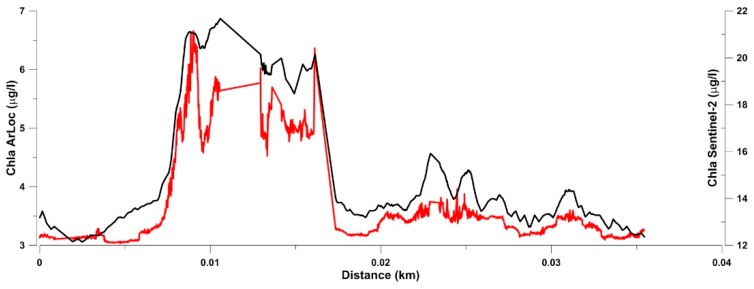
Normalized chlorophyll *a* trend performed in a representative transect along Blomstrandbreen glacier, (red for ArLoC data and black for Sentinel-2 data).

The ArLoC trend line matches the satellite-derived product satisfactorily (Figure 10), showing a good correspondence (r^2^ = 0.926) mainly in the zone where geographical variations are more significant. However, this result is preliminary: It represents a first step both towards accurate validation of satellite data in these extreme areas and towards applying the best parametrization for the selected zone.

## 4. Discussion and Conclusions

The ArLoC (Arctic Low-Cost probe) is a cost effective, flexible probe developed for efficient integration into different kinds of oceanographic platforms. This probe is constituted by a flow-through chamber equipped with depth, temperature and chlorophyll *a* fluorescence sensors. Its modular mechanical components make it possible to change its configuration easily. Despite their low cost, the ArLoC sensors have proved to be effective in maintaining a high resolution, when compared with moderately expensive commercial sensors and probes. Several instruments are available for marine measurements, but in general even middle-range probes offer a lower resolution than ArLoC’s. In terms of expenditure, ArLoC can be considered a low-cost item, whereas in terms of resolution it performs like a middle-range/top quality probe.

If this kind of technology became readily available, the capacity of observational networks would improve significantly, by expanding the data sets that could be acquired in support of operational oceanography and remote sensing image validation.

To test ArLoC’s performance, an experimental survey took place in the Kongsfjorden area in summer 2017. The ArLoC was installed onboard an unmanned marine vehicle, PROTEUS (Portable RObotic TEchnology for Unmanned Surveys), which was also equipped with ‘state of the art’ probes, such as Idronaut 305 plus. The PROTEUS technology, which proved its effectiveness during the campaign, is based on the concepts of portability, modularity and reconfigurability. In parallel, the use of a new sensor such as ArLoC (a light, modular and easy-to-integrate instrument) increases the efficiency of robotic tools in harsh environments with complex logistics.

Comparison between data acquired by ArLoC and by the ‘state of the art’ probe revealed strong consistency. The results confirmed the accuracy of the ArLoC temperature sensor when compared with the Idronaut 305 plus, a much more expensive probe. Chlorophyll *a* data also matched the local concentrations [7,31,32,33]. In this study, ArLoC allowed us to investigate, at high spatial resolution, chlorophyll *a* distribution near the Blomstrandbreen glacier during ice melting. The results highlight a high biomass concentration in proximity of the glacier front (featuring colder water masses), thus confirming the influence of ice melting on phytoplankton bloom. 

Moreover, this test underlines the exceptional capacity of the PROTEUS platform to detect microscale ecological processes in extreme environments, such as the Arctic Ocean, where natural hazards (e.g., icebergs, falling masses of ice, etc) hinder normal sampling activities. The study of these phenomena is of fundamental importance in increasing our knowledge of global warming process, which directly affect ice melting. In turn, this leads to an increase in continental inputs to surface waters, resulting in phytoplankton growth.

This work has described preliminary tests carried out to verify if the ArLoC probe can be used to validate the algorithms applied to high resolution remote sensing observations. Chla concentration measured by ArLoC was compared with the product obtained by applying the empirical algorithm provided by Tzortziou et al. [37] to Sentinel-2, which is suitable for detecting ecological processes occurring near Blomstrandbreen glacier. The comparison between the two datasets showed that ArLoC is quite accurate in reproducing the chla variations calculated from satellite data, confirming that a low-cost but high-resolution technology may perform remarkably well in validating satellite algorithms, especially in extreme environments where measuring implementation is often a big challenge. The promising results obtained in this experiment, although preliminary, pointed out that the spatial variations are strongly correlated, even if a larger data set is necessary to improve the current algorithm’s performance.

To improve future perspectives in extreme environment monitoring, deeper measurements should also be carried out (and in fact, activities were programmed to develop and test instruments for harsher conditions), enabling the study of subsurface and deep structures (for instance Svalbard phytoplankton behavior and distribution connected to ice melting). Knowledge of these phenomena will help enhance comprehension of the impact of temperature rising on this sensitive polar region which plays a key role in global climate change.

## Figures and Tables

**Table 1:** Main specifications of commercial marine sensors and probes.

Sensor-Probe	Measure	Accuracy-MDL *	Resolution
SBE 37 MicroCAT ^(a)^	Temperature (CTD)	±0.002 °C	0.0001 °C
YSI 600OMS ^(b)^	Temperature (Multiparametric)	±0.15 °C	0.01 °C
Idronaut Ocean Seven CTD ^(c)^	Temperature (CTD)	±0.0015 °C	0.0001 °C
Cyclops-7F Turner Design ^(d)^	Chla Fluorescence	0.03 µg/L	-
ECO FL Wetlabs ^(e)^	Chla Fluorescence	0.02 µg/L	-
SeaPoint ^(f)^	Chla Fluorescence	0.02 µg/L	-
UniLux Chelsea ^(g)^	Chla Fluorescence	0.01 µg/L	-
MicroFlu-chl Trios ^(h)^	Chla Fluorescence	0.02 µg/L	
EXO1 YSI ^(i)^	Chla Fluorescence (Muliparametric)	0.01 µg/L	-
ArLoC	Temperature	±0.01 °C	0.001
	Chla Fluorescence	0.01 µg/L	-

* Minimum Detectable Limit; ^(a)^ http://www.seabird.com/sbe37sm-microcat-ctd; ^(b)^ https://www.ysi.com/600OMS-V2; ^(c)^ http://www.idronaut.it/cms/view/products/multiparameter-ctds/environmental-ctds/ocean-seven-310/s298; ^(d)^ http://www.turnerdesigns.com/t2/doc/manuals/998-2100.pdf; ^(e)^ http://www.seabird.com/wetlabs; ^(f)^ http://www.seapoint.com/scf.htm; ^(g)^ https://www.chelsea.co.uk/products/marine-science/fluorometers/unilux-fluorometer; ^(h)^ http://www.spectrasens.com/Waterkwaliteit/CHL_MicroFlu_manual%20ver%201_2.pdf; ^(i)^ https://www.ysi.com/EXO1.
